# Drug-Related Pneumonitis in Cancer Treatment during the COVID-19 Era

**DOI:** 10.3390/cancers13051052

**Published:** 2021-03-02

**Authors:** Sara Cherri, Silvia Noventa, Martina Fanelli, Giulio Calandra, Tiziana Prochilo, Claudio Bnà, Giordano Savelli, Alberto Zaniboni

**Affiliations:** 1 Unit of Medical Oncology, Department of Oncology, Fondazione Poliambulanza, 25124 Brescia, Italy; silvia.noventa@poliambulanza.it (S.N.); tiziana.prochilo@poliambulanza.it (T.P.); alberto.zaniboni@poliambulanza.it (A.Z.); 2 Medical Oncology Unit, University Hospital of Modena, 41124 Modena, Italy; martinafun89@gmail.com; 3 Unit of Radiology, Department of Diagnostic Imaging, Fondazione Poliambulanza, 25124 Brescia, Italy; giulio.calandra@poliambulanza.it (G.C.); claudio.bna@poliambulanza.it (C.B.); 4 Nuclear Medicine Department, Fondazione Poliambulanza, 25124 Brescia, Italy; giordano.savelli@poliambulanza.it

**Keywords:** lung toxicity, interstitial lung disease, pneumonitis, cancer treatment, target therapy, chemotherapy, immunotherapy, COVID-19

## Abstract

**Simple Summary:**

Interstitial lung disease is a group of diseases characterized by chronic lung inflammation that can be related to oncological treatments, such as traditional chemotherapy drugs and the newest targeted therapies and immunotherapies. Awareness about this potentially fatal adverse event is paramount in patient management and to make a conscious therapeutic choice. It represents a differential diagnostic challenge, especially in the context of the COVID-19 pandemic. Our aim is to describe the incidence and characteristics of this adverse event across oncological treatment groups and to promote greater knowledge about this important toxicity.

**Abstract:**

Interstitial lung disease is recognized as a group of diseases with a different etiopathogenesis characterized by chronic lung inflammation with the accumulation of inflammatory cells, lymphocytes and macrophages, and the consequent release of proinflammatory cytokines. Various degrees of pulmonary fibrosis can be associated with this inflammatory condition. Interstitial lung disease related to oncological drugs is a relevant problem in clinical practice. The etiopathogenetic mechanisms underlying this adverse event are not completely known but can be partly explained by the mechanism of action of the drug involved. Therefore, knowledge of the relevance of this potentially fatal adverse event supported by the reported safety data of pivotal studies becomes fundamental in the management of patients. The prompt diagnosis of drug-related pneumonia and the consequent differential diagnosis with other forms of pneumonia allow a rapid suspension of treatment and the establishment of an immunosuppressive treatment if necessary. In the context of the health emergency related to SARS CoV2 infection and COVID-19-related interstitial lung disease, such knowledge holds decisive relevance in the conscious choice of cancer treatments. Our intent was to describe the oncological drugs most correlated with this adverse event by reporting, where possible, the percentages of insurgency in pivotal studies to provide an overview and therefore promote greater awareness of this important toxicity related to oncological treatment.

## 1. Introduction

On 9 January 2020, a new coronavirus, SARS-CoV-2, was defined as the causative agent of a cluster of pneumonia cases reported in Wuhan, China. The disease caused by the new coronavirus was named COVID-19. On 11 March 2020, the World Health Organization (WHO) Director General indicated that the spread of COVID-19 was no longer an epidemic confined to certain geographical areas, but a pandemic spread throughout the planet. Thus, a dramatic period began worldwide that led to the collapse of the health system as we know it. Within this context, medical oncology has had to question the benefit–risk ratio of cancer treatments during the pandemic not only because of the increased risk of contagion for patients when going to the hospital to receive cancer treatments, but also because of worrying data about the impact of cancer on the prognosis of SARS-CoV-2 infection [1].

SARS-CoV-2 infection does not present with the same severity in all patients, in particular, three potential scenarios are recognized: mild, severe and critical illness. In mild disease, mild pneumonia or no pneumonia develops, and usually in this form, symptoms of viral upper respiratory tract infection prevail. Conversely, critical illness is characterized by severe pneumonia with a systemic presentation and potential multiorgan failure. X-ray images are characterized by the presence of interstitial lung disease with uneven ground glass opacities and uneven consolidation in the intermediate, outer and subpleural areas of the lung [2].

Many cancer drugs can also cause interstitial lung damage [3]. Pulmonary toxicities are not among the most common adverse events of drugs used in the treatment of solid tumors; however, there are no clear data on their incidence. In this context, case reports and real-life data can make a valuable contribution to better quantify the impact of this toxicity, which in some cases can be fatal. It is important to take this toxicity into account in the decision-making process and, second, to suspect drug-related interstitial lung disease in case of suggestive symptoms to promptly suspend the therapy when necessary. Therefore, the choice of oncological treatment during the era of COVID-19 can be difficult. We conducted a qualitative review of the evidence in the literature regarding pulmonary toxicity from oncological drugs, focusing in particular on immunotherapy, target therapy and conjugated antibodies recently approved by the FDA (Food and Drug Administration) and EMA (European Medicines Agency) for the treatment of solid tumors with the aim of understanding the relevance of this issue.

## 2. Chemotherapy and Lung Toxicity

Interstitial pneumonitis related to chemotherapy drugs represents a relevant oncological problem that has been investigated both in preclinical and clinical studies to understand the underlying mechanisms. Pulmonary toxicity of some of the older chemotherapy standard drugs may be dose-dependent, i.e., bleomycin [4], or it can be observed several years after their use, i.e., cyclophosphamide [5]. To date, there are no tools to prevent the onset of interstitial pneumonitis other than the careful clinical evaluation of patients who develop respiratory symptoms and radiological monitoring in the most fragile subjects. Table 1 presents the main chemotherapy drugs that can lead to the development of interstitial pneumonia.

Bleomycin is a chemotherapy drug that is classically related to such toxicity, as noted in the first clinical trials in the 1960s. A prevalence of pulmonary toxicity of 40–45% has been reported with regimens including bleomycin, with a fatal outcome in 1–3% of these cases. There is currently no protocol to prevent this adverse event; however, some risk factors are recognized, such as the cumulative dose of bleomycin, reduction in glomerular filtration rate (GFR), elevated creatinine and advanced age [6]. With regard to the mechanism of lung damage induced by bleomycin, a possible interpretative key is oxidative damage, which could lead to pulmonary toxicity in subjects with deficiency of the enzyme that physiologically deactivates bleomycin hydrolase, leading to the release of inflammatory cytokines and consequent pulmonary fibrosis [7]. Bleomycin hydrolase, an enzyme that degrades bleomycin, is active in all tissues except for skin and lung, partly explaining the drug’s specific toxicity to these organs. Studies examining the different susceptibilities of mouse strains to bleomycin reveal that a bleomycin-resistant strain produces considerably more bleomycin hydrolase than a bleomycin-sensitive strain. The acute lung damage observed in bleomycin-sensitive mice was attributed to DNA strand splitting, resulting in chromosomal damage. The chronic fibrotic response to bleomycin-induced damage is associated with an acquired loss of bleomycin hydrolase activity and is mediated by the migration of activated effector cells into the lung with the release of proinflammatory mediators. Nude (athymic) mice are resistant to bleomycin-induced lung damage, suggesting that the inflammatory process is necessary for the pathogenesis of the disease. Proinflammatory lung injury mechanisms, similar to those reported in bleomycin studies, are also observed with methotrexate and cyclophosphamide [8,9]. Studies have demonstrated that in response to excess free radicals resulting from methotrexate use, an interleukin response may be induced with activation of p38 mitogen-activated protein kinases (MAPK p38), kinases involved in pulmonary inflammation and fibrosis [10,11]. In the case of cyclophosphamide, inflammatory processes are also described with an increased inflammatory cascade (TGFβ, fibronectin and procollagen) in response to DNA damage and oxidative stress.

Despite a reduced incidence, drug-related reports of interstitial pneumonia also involve other widely used chemotherapeutic drugs, such as platinoids, taxanes and gemcitabine (see Table 1). With regard to gemcitabine, a systematic review on severe lung toxicity reported an incidence of up to 5% [12]. The clinical presentation is mostly nonspecific and requires a diagnosis of exclusion from other causes of interstitial pneumonitis. The predominant radiographic pattern is represented by reticulo-nodular interstitial infiltrates. It has been postulated that the pathophysiological mechanism of gemcitabine-mediated lung injury is a cytokine-mediated inflammatory reaction of the capillary-alveolar walls, with consequent alteration of membrane permeability [13]. For platinoids, when used alone, pulmonary toxicities are very rare, if not anecdotal. On the other hand, interstitial pneumonia associated with the use of taxanes is of greater relevance, with an incidence of grade 3 or higher in approximately 1% to 5% of patients receiving paclitaxel or docetaxel at conventional doses thrice weekly [14]. As previously mentioned, an increased risk is reported in the combined treatments [15].

## 3. Target Therapies and Lung Toxicity

This list of target therapies leading to ILD is certainly destined to extend the updated safety data of drugs that have been used in clinical oncology for the past few years [42]. For example, the FDA recently published updated safety data for the cyclin inhibitor drugs palbociclib, ribociclib and abemaciclib, warning that these drugs can cause rare but serious pneumonitis [43]. This topic will also deserve careful consideration in the coming years given that this type of toxicity was reported for drugs not yet approved in Europe but that are likely to be available in the next few years. Table 2 reports target therapies causing lung injury.

### 3.1. Tyrosine Kinases Inhibitors

Tyrosine kinase inhibitors (TKIs) are small molecules that inhibit the activation of protein kinases that, by means of protein phosphorylation, are involved in the activation mechanisms of proteins involved in cell growth processes. These proteins are often hyper-expressed or hyperactivated in some forms of cancer, making these drugs a very important therapeutic weapon in the treatment of solid tumors. Table 2 presents the TKIs for which cases of drug-related interstitial pneumonia were reported in the technical data sheet and registration studies.

The underlying mechanism of pulmonary toxicity can be different from one molecule to another. However, the mechanism is not completely known and partly depends on the mechanism of action of the drug itself. For example, it was postulated that interstitial lung disease caused by gefitinib, an EGFR inhibitor used in the treatment of EGFR-mutated non-squamous NSCLC, is most likely related to a decrease in alveolar regeneration, a process normally regulated by EGFR, in a population with a high co-incidence of lung disease [44]. In a meta-analysis conducted to define the different toxicity profiles of EGFR TKI, erlotinib, gefitinib and afatinib, toxicity-related deaths were rare (1.7%), with pneumonitis being the most frequent cause and no significant difference between the different drugs [44]. Another meta-analysis conducted to evaluate the risk of ILD associated with the use of EGFR-TKIs, gefitinib, erlotinib and afatinib, concluded that the incidences of all-grade and high-grade ILD were 1.6% and 0.9%, respectively. Again, no significant difference in ILD risk was found in the subgroup analysis by EGFR-TKI drugs [45]. For osimertinib, the latest generation of anti-EGFR TKIs, in the pivotal study FLAURA, 4% of patients in the osimertinib arm developed interstitial lung disease compared to 2% of patients in the standard EGFR-TKI arm (gefitinib or erlotinib) [46].

ALK inhibitors, either first (crizotinib), second (alectinib, ceritinib, brigatinib) or third generation (lorlatinib), can also lead to lung interstitial toxicity. Several hypotheses have been suggested to better clarify the mechanisms responsible for pulmonary toxicity exerted by ALK-TKIs with particular regard to crizotinib, but they all derive from case reports and retrospective studies [51]. In a systematic review conducted by Pellegrino and colleagues, pulmonary adverse events attributed to crizotinib occurred in 1.8% of cases, 1.1% attributed to ceritinib, 2.6% to alectinib, 1.8% to lorlatinib and 7% to brigatinib, which was therefore identified as the ALK inhibitor with the highest percentage of ILD [47]. Across all studies of brigatinib, at a starting dose of 90 mg once daily, 4.5% of patients experienced any grade event of pneumonitis with a median time to onset of 2 days. Three percent of patients had grade 3 or higher events that led to drug discontinuation [48,51]. In a recent study by Hwang et al. in patients with NSCLC treated with ALKs, COP was the most common pattern (64% of the sample), followed by HP and AIP. All patients with COP were successfully cured, whereas half of them with AIP died [85].

ILD during treatment with anti-HER2 tyrosine kinase inhibitors is rarely reported in the literature. Four studies totaling 4470 patients who received lapatinib were reported in a recently published review, eight of whom (0.2%) had at least one reported ILD event [52]. Tucatinib is a highly selective oral tyrosine kinase inhibitor for the kinase domain of HER2, with minimal inhibition of the epidermal growth factor receptor. In the pivotal study, Her2climb, a percentage of 1.2% of patients in the tucatinib arm reported ILD [53]. Several antiangiogenic TKIs with different binding capacities to angiogenic kinases are used in clinical practice: sorafenib, sunitinib, pazopanib, regorafenib, cabozantinib, nintedanib and axitinib. It is important to remember that these molecules act not only on vascular endothelial growth factor receptor (VEGFR) but also on multiple kinase receptors. For example, cabozantinib is a multi-kinase inhibitor of VEGFR-1, -2 and -3, KIT, TRKB, FLT-3, AXL, RET, MET and TIE -2, and regorafenib exhibits a broad spectrum of activity with inhibition of tyrosine kinases involved in tumor angiogenesis mechanisms (e.g., PDGFR, FGFRs 1–2, VEGFRs 1–3, TIE2), proliferation (RET, RAF, KIT), tumor microenvironment and metastasis processes (VEGFR2–3, PDGFR).

When used alone, adverse pulmonary events are rare and mostly described in case reports. ILD is more common when a multi-kinase inhibitor also inhibits platelet-derived growth factor receptor (PDGFR). A PDGFRα and PDGFRβ inhibitor associated with ILD, although rarely, is imatinib [58]. A possible mechanism of lung injury could be related to PDGFR inhibition. Imatinib-induced ILD is likely to develop in more susceptible patients, such as those with a history of pneumonia. However, ILD occurs less frequently with VEGFR and PDGFR-TKIs than with EGFR-TKIs. Sorafenib in particular has rarely been associated with cryptogenic organizing pneumonia (COP) and non-specific interstitial pneumonia (NSIP) patterns [55].

Another class of TKIs with great clinical relevance in oncological treatments, especially melanoma, non-small-cell lung cancer (NSCLC) and, recently, colorectal cancers, is BRAF and MEK tyrosine kinase receptor inhibitors. Respiratory complications are extremely rare with BRAF inhibitor monotherapy, such as vemurafenib or dabrafenib. Regarding mek-inhibitors (MEKi), in the pivotal trametinib + dabrafenib doublet study conducted by Flaherty and colleagues [59], interstitial pneumonia occurred in 2.4% of patients treated with trametinib alone, and all patients who presented this adverse event required hospitalization. The median time to first presentation was 160 days (range 60–172 days). Therefore, in all patients treated with trametinib who present with cough or suspicious symptoms and signs of pneumonitis, radiological examinations and temporarily suspended treatment should be investigated [59].

### 3.2. mTOR

Regarding mTOR inhibitor (mTORi) drugs, ILD is a widely described and studied adverse event with high relevance and incidence in clinical practice. Although the majority of patients with mTORi-related ILD are asymptomatic or mildly symptomatic, it is important not to underestimate this clinical finding and to adhere to the recommendation regarding the management of this toxicity, as in some cases, it can lead to important respiratory outcomes. All mTORi drugs can be related to this adverse event; however, among them, the pulmonary toxicity profile appears to differ. A retrospective analysis of 196 patients revealed a higher incidence of ILD in patients receiving everolimus than in those receiving temsirolimus (38% vs. 22%, *p* = 0.018) [63]. The reported incidence of mTORi pulmonary toxicity in the literature differs from that of clinical studies, probably due to a greater awareness of this adverse event and greater precision in radiological diagnostic criteria. The first phase 2 clinical trials with everolimus and temsirolimus reported incidences of clinically manifest ILD of 3–13%. Real-life studies report an incidence of ILD that ranges from 14% to 45% for temsirolimus and from 3% to 54% for everolimus [86,87]. Regarding the mechanisms of lung damage, unlike bleomycin, a dose-dependent correlation is not evident [67]. The pathophysiological mechanisms of mTORi-mediated lung injury are complex. Preclinical studies have described direct damage to the endothelium and epithelium, depletion of alveolar macrophages and accumulation of surfactant lipids. Both epithelial and endothelial damage with accumulation of surfactant contribute to a pulmonary inflammatory state. Furthermore, mTORi-mediated epithelial damage could expose cryptic antigens, activating a T-mediated immune response, resulting in lymphocytic alveolitis and interstitial pneumonia [88]. It is also possible that the mTORi molecule leads to a delayed-type hypersensitivity reaction. Indeed, mTORi has a high affinity for plasma proteins, and the resulting mTORi-protein complex can be immunogenic, processed by antigen-presenting cells and recognized by T cells. This process leads to cytokine release and preferential differentiation of Th0 cells into Th1 cells with recruitment and activation of macrophages and other inflammatory cells. Finally, several preclinical studies have demonstrated how mTOR inhibitors interfere with the pathways of damage and repair of lung tissue by inducing a sustained inflammatory response through the downregulation of the phosphatidylinositol-3-kinase (PI3K) pathway and the consequent production of pro-cytokines, such as interleukin (IL)-12, IL-23, tumor necrosis factor (TNF) and IL-1β [89]. Again, clinically, the symptoms and signs are nonspecific. The time to onset of ILD after initiation of treatment is relatively short, mostly occurring within 6 months after starting treatment.

A diagnosis of noninfectious pneumonitis should be considered in patients presenting with nonspecific respiratory signs and symptoms, such as cough, dyspnoea, hypoxia, fever and fatigue, and in whom infectious, neoplastic and other nonmedicinal causes have been excluded by means of appropriate investigations. Patients who develop radiological changes and have few or no symptoms may continue everolimus without dose adjustments (Grade 1). If symptoms are moderate (Grade 2) or severe (Grade 3), the drug should be discontinued, and the use of corticosteroids (such as oral prednisone 0.75–1.0 mg/kg or in severe cases, intravenous methylprednisolone) may be indicated until clinical symptoms resolve. Given the immunosuppressive properties of everolimus, the use of corticosteroids and possible respiratory distress, the coadministration of broad-spectrum antibiotics may also be considered for Grade 3 or 4. Opportunistic infections should also be considered; indeed, prophylaxis for *Pneumocystis jirovecii* (carinii) pneumonia is recommended [90].

Generally, in mTOR inhibitor pneumonitis, pulmonary function tests are associated with a restrictive pattern or an isolated reduction in diffusing capacity [91]. The most characteristic radiological changes include ground-glass and reticular opacities, in particular of the lower lobes of the lungs. mTOR inhibitor-associated pneumonitis most commonly presents as either cryptogenic organizing pneumonia (COP) or nonspecific interstitial pneumonia (NSIP) [92,93].

Some evidence suggests that everolimus-related pneumonia is associated with improved prognosis and may be used as a biomarker for the efficacy of the drug, especially in breast cancer [94].

### 3.3. Phosphatidylinositol 3-Kinase (PI3K) Inhibitors

Activation of the PI3K signaling pathway is frequently associated with tumorigenesis. Moreover, dysregulated PI3K signaling may contribute to tumor resistance to a variety of cancer drugs. Several molecules that act on the Pi3k-AKT signaling pathway are being studied. A PIK3 inhibitor approved by the FDA and EMA for the treatment of solid tumors is alpelisib. This new molecule has recently entered clinical practice, and the possible range of side effects is not completely known. Pneumonitis occurred in 1.8% of patients receiving alpelisib in the pivotal SOLAR-1 study [62]. Specific monitoring guidelines have been established to address this side effect. In particular, permanent discontinuation of alpelisib is advised in any patient who develops pneumonitis [95].

### 3.4. Monoclonal Antibodies

Monoclonal antibodies used in clinical practice in the treatment of solid tumors are monoclonal antibodies against Her family receptors, such as panitumumab, cetuximab and trastuzumab, and monoclonal antibodies that target vascular endothelial growth factor (VEGF), such as bevacizumab, or its receptor, such as ramucirumab.

For monoclonal antibodies targeting Her family receptors, panitumumab and cetuximab bind directly to EGFR, whereas trastuzumab and pertuzumab bind to the HER2 protein expressed on the cell surface, inhibiting the EGFR pathway. Unlike tyrosine kinase inhibitors acting on EGFR, for which ILD is a widely described adverse event, its incidence is not known for monoclonal antibodies, and reports of this event are very rare. In pivotal studies of panitumumab and cetuximab, interstitial pneumonia was not a reported event. Regarding trastuzumab, in a pivotal study in the adjuvant setting, the rate of interstitial pneumonitis was 0.6% [71]. COP is the prevailing high resolution computed tomography (HRCT) pattern [96]. A review of the literature conducted with the aim of determining the incidence of ILD in patients undergoing anti-HER treatments showed 162 cases (9.9%) of drug-related ILD. Overall, there were 3 (0.2%) ILD-related deaths among those receiving trastuzumab therapy [55]. In the EMA datasheet for pertuzumab, ILD is reported as a rare event; to our knowledge, there are no case reports for pertuzumab concerning this topic. In the CLEOPATRA combination study of docetaxel, pertuzumab and trastuzumab in the metastatic first-line Her2+ breast cancer, no significant rates of pneumonia were reported compared to the docetaxel and trastuzumab placebo arms, which occurred in a very low percentage of patients [72].

For monoclonal antibodies targeting VEGF or its receptor (VEGFR), interstitial pneumonitis is a very rare event and mostly reported as case reports. A study on bevacizumab, a monoclonal antibody directed at VEGF, hypothesized a protective role of bevacizumab on interstitial pulmonary toxicity mediated by chemotherapy; however, there are no data in the literature to support this hypothesis [76]. Ramucirumab is a VEGFR-2 inhibitor. In the phase 3 RAINBOW pivotal trial of ramucirumab in combination with paclitaxel in second-line treatment of advanced gastric cancer, the incidence of treatment-related pneumonitis was lower in patients who received the combination treatment (1.5%) than in those who received paclitaxel alone (2.1%) [77]. In the REGARD study of ramucirumab monotherapy in pretreated advanced gastric cancer, ramucirumab-related pneumonia occurred in 0.4% of patients [78]. In a retrospective study of 44 gastric cancer patients who received combination treatment with ramucirumab and paclitaxel, six patients (13.6%) developed pneumonitis during the first five cycles of treatment. The onset of pneumonitis was independently associated with the presence of pre-existing ILD (*p* = 0.025; odds ratio = 206.4) [79].

### 3.5. Antibody-Drug Conjugates (ADCs)

Antibody-drug conjugates (ADCs) are complex molecules composed of a monoclonal antibody bound to a biologically active cytotoxic drug, thus combining the ability to “target” specific molecules, typical of monoclonal antibodies, with the cytotoxic properties of chemotherapy drugs. Among the ADCs are trastuzumab emtansine and a series of new drugs recently approved by the FDA but still under approval in Europe for the treatment of solid tumors, namely, enfortumab vedotin, trastuzumab deruxtecan and sacituzumab govitecan. The pulmonary toxicity profile is very different for this class of drugs.

Trastuzumab emtansine (T-DM1) is an ADC that combines the monoclonal antibody trastuzumab with cytotoxic mertansin (DM1), a maytansinoid class anti-microtubule agent bound by a stable thioether. T-DM1 binds to the HER2 receptor, and the HER2 and T-DM1 complex enters target cells through receptor-mediated endocytosis. This process leads to the intracellular release of DM1, favoring its cytotoxic activity. In addition, the drug retains its anti-HER2 properties, including inhibition of HER2 intracellular signaling pathways and induction of cell-mediated cytotoxicity. Cases of ILD have been reported in patients receiving T-DM1. Pneumonitis of any grade shows an incidence rate up to 9%, whereas severe pneumonitis (grade ≥ 3) was recorded in 1–6% of all patients treated with T-DM1 [80].

Enfortumab vedotin is a human IgG1 antibody directed against nectin-4, an adhesion protein located on the cell surface. The small molecule MMAE is an antimitotic agent that interferes with the formation of microtubules attached to the antibody via a clearable linker with protease. Additionally, in this case, internalization through the ligand allows the cytotoxic activity of the drug. In a pivotal study of enfortumab vedotin in urothelial carcinoma after treatment with platinum and immunotherapy, one treatment-related death from ILD was reported [81].

Sacituzumab govitecan is an anti-Trop-2 antibody conjugated with SN-38 Trop-2, an active metabolite of irinotecan. Irinotecan is a chemotherapeutic agent that has been associated with cases of interstitial pneumonia, so it is plausible that sacituzumab govitecan may present this toxicity as well. However, in the phase 1–2 study of sacituzumab govitecan in patients with metastatic triple-negative breast cancer, no cases of ILD were observed among the 108 patients enrolled. Among the 5 patients (5% of total) who experienced Grade 3 or 4 adverse respiratory events, none of the events were considered by the investigators to be related to sacituzumab govitecan. More data will certainly be needed to understand whether the drug may present such toxicity [82].

Trastuzumab deruxtecan is an ADC where the antibody directed against HER-2 is conjugated to a topoisomerase inhibitor. In the single-arm study DESTINY-Breast01, ILD was reported in 13.6% of patients receiving trastuzumab deruxtecan, leading to death in 2.2% of patients [83,84]. Numerous ongoing trials are evaluating trastuzumab deruxtecan in the treatment of different tumor histotypes. Certainly, the problem of pulmonary toxicity will be of extreme importance in the near future, in which the rapidly increasing therapeutic scenario sees these drugs as the main leading actor in the target treatment across tumor types.

## 4. Immunotherapy and Lung Toxicity

Immunotherapy treatment has become the standard of care in the metastatic setting in several neoplastic diseases. Since the first immunotherapy studies, the problem of interstitial pulmonary toxicity has been highlighted as an important side effect to be taken into consideration. ILD resulting from immunotherapy drugs recognizes a clear pathophysiological mechanism linked to the activity of the drug itself.

Programmed death-1 (PD1), the PD-1 ligand (PDL1) and the cytotoxic T-4 lymphocyte-associated antigen (CTLA4) are called immune checkpoint molecules because they negatively regulate host immunity. This ability to evade immune surveillance is part of the tumor skipping and disease growth process. Immune checkpoint inhibitors are antibodies that inhibit PD-1, PD-L1 or CTLA-4, resulting in activation of the immune response. The toxicity profile of immunotherapy drugs differs from that of cytotoxic drugs and is characterized by peculiar adverse events, known as immune-related adverse events (irAEs) [97]. Immune-mediated interstitial pneumonia is rarer than other irAEs but more severe [98].

The incidence of this toxicity is higher in patients undergoing immunotherapy treatment for lung cancer than in other tumor types reported for targeted therapies is higher in patients with a history of previous pulmonary diseases [99]. It is important to emphasize that combination therapies with anti-PD-1 and anti-CTLA4 antibodies show a higher incidence of ILD than monotherapy. To date, no identifiable risk factors for ILD are known that could be considered to prevent its onset. A single-center retrospective study conducted by Okada and colleagues among 102 patients treated for lung cancer diagnosed with ILD concluded that ECOG PS (Performance Status according to Eastern Cooperative Oncology Group) ≥ 2 alone or the presence of both ECOG PS ≥ 2 and a history of smoking with ≥50 pack-years acted as risk factors for Grade ≥ 3 and all grades of ILD, respectively [100]. In descending order of toxicity, the most reported patterns are AIP (high grade), COP (intermediate grade) and finally, NSIP and HP (low grade) [101].

Several literature reports propose the onset of irAEs, such as interstitial pneumonitis, as a predictor of response to immunotherapeutic drugs, but this assumption is not currently considered certain [102]. Table 3 reports the immunotherapy drugs currently used in clinical practice classified by the mechanism of action (anti-PD1, anti-PDL1 and anti-CTLA4), their indication in the various neoplastic pathologies and the relative incidence of interstitial pneumonitis reported in pivotal studies.

### 4.1. Anti-PD1

PD-1 is a member of the immunoglobulin superfamily that can be detected on activated T cells, B cells and natural killer (NK) cells, and binds PDL-1 and PDL-2. PD-L1, expressed by tumor cells and immune cells, also interacts with CD80, whereas PD-L2, expressed only on dendritic cells in normal tissue, interacts with RGMb (repulsive guide molecule B). All these interactions mediate an inhibitory signal that leads to the suppression of T cell activation. Anti-PD1-related lung toxicity, including ILD, recognizes autoimmune genesis following activation of T cells. Patients treated with PD1 antibodies should be monitored for signs and symptoms of pneumonia, clinical suspicion should be confirmed with radiographic images and other potential causes must be excluded [132]. In cases of Grade ≥ 2 pneumonia, immunotherapy is temporarily suspended, and steroid treatment is administered with an initial dose equivalent to 1–2 mg/kg/day prednisone. Instead, treatment should be permanently discontinued for Grade 3, Grade 4, or Grade 2 recurrent pneumonia. The anti-PD-1 monoclonal antibodies approved by the FDA and EMA for the treatment of solid tumors include nivolumab and pembrolizumab.

The incidence of pneumonia in pivotal studies of nivolumab monotherapy ranges from 1.3% to 5% and is higher in patients with NSCLC [107,108]. Regarding the combination of nivolumab with ipilimumab, an anti-CTLA4, in melanoma, the incidence of pneumonia was 6.4% [110]; however, in RCC, the incidence was 6.2% [111]. Pembrolizumab is an anti-PD 1 approved for the treatment of solid tumors both alone and in combination with chemotherapy. Keynote 426 enabled the registration of pembrolizumab in combination with axitinib as the first-line renal cell carcinoma treatment. In the pembrolizumab studies in NSCLC, the rate of interstitial pneumonia was higher than in the other studies, especially in patients with a history of chest radiation therapy (see Table 3).

### 4.2. Anti-PDL1

Another class of immune checkpoint inhibitors are monoclonal antibodies that inhibit PDL-1. As mentioned, activation of PD-1/PD-L1 signaling negatively regulates T cell-mediated immune responses in peripheral tissues. Through antibody-mediated PD-L1 inhibition, the immunosuppressive signals present in the tumor microenvironment are reduced with a consequent increase in T cell-mediated immunity against tumors [133]. Currently, there are three FDA- and EMA-approved PD-L1 inhibitors for the treatment of various malignancies, namely, atezolizumab, durvalumab and avelumab. Table 3 shows their current indications in clinical practice and the percentage of pneumonia reported in pivotal studies.

Special mention should be made of the Pacific study, which led to the approval of durvalumab in locally advanced, unresectable non-small cell lung cancer patients with PD-L1 expression ≥ 1% whose disease did not progress after platinum-based chemoradiotherapy [126]. As also reported for other oncological drugs, including chemotherapy, biology and immunotherapy, the percentage of interstitial pneumonia is higher in patients undergoing radiotherapy on the chest [134,135]. Therefore, a careful clinical evaluation of patient candidates to receive durvalumab in this setting is strongly recommended.

### 4.3. Anti-CTLA4

CTLA-4 is a coinhibitory molecule and is the counterpart of the costimulatory B7-CD28 axis. Upon activation, T lymphocytes positively regulate the surface expression of CTLA-4, which binds B7 with increased avidity and thereby overcomes the positive costimulatory signal of CD28. This dominance of negative signals results in a reduced proliferation of T cells and a decrease in the production of IL-2. The blockade of CTLA-4, and therefore the release of B7 for the interaction with the costimulatory molecule CD28, activates the immune response [136].

Anti-CTLA4 monoclonal antibodies exhibit a higher rate of immune-related adverse events, including pneumonitis, compared with anti-PD1 and anti-PDL1 drugs. In the CheckMate 238 study, which randomized patients to receive nivolumab or ipilimumab as adjuvant treatment in resected stage III or IV melanoma, pneumonia occurred in 1.3% of cases in the nivolumab arm and in 2.4% in the ipilimumab arm [104]. The mechanisms underlying the higher rate of immune-related side effects from anti-CTLA-4 drugs remain unclear. Currently, their use as monotherapy in clinical practice remains confined to melanoma [130].

## 5. Diagnosis and Therapy

Several radiological patterns are used to describe interstitial lung disease (ILD), unfortunately mostly without specificity to differentiate the multitude of potential conditions, which include infections (especially viral), immunological diseases (also allergies), toxicities, idiopathic forms and even possibly mimic tumor progression. Radiology can be useful in grading the severity and guiding therapeutic management. As defined by the American Thoracic Society and the European Respiratory Society (ATS/ERS) in correlation with histological findings, the most common patterns are presented below [137]. It is worth noting that it may occasionally not be possible to identify a case with a specific pattern, and even overlapping among them is possible [138].

AIP (acute interstitial pneumonia) has been historically associated with bleomycin, alkylating agents (such as cyclophosphamide) and antimetabolites (such as methotrexate). Extensive ground-glass opacities and dependent consolidations can be found in a setting that can correspond to ARDS (acute respiratory distress syndrome). Fibrosis may develop within one week, and the prognosis is mainly poor. Corticosteroid therapy is recommended.

NSIP (non-specific interstitial pneumonia) is typically characterized by subtle evolution, distinguishable into cellular and fibrotic types, and is correlated with more indolent and aggressive clinical forms. Ground-glass opacities are the dominant feature, displaying basal predominance with sparing of subpleural spaces. Thickening of bronchovascular bundles with traction bronchiectasis and consequently lung volume loss are the expressions of the advanced stage. Early diagnosis can significantly impact therapeutic and prognostic responses that depend on the extent of fibrosis. Mycophenolate mofetil can improve lung function. Methotrexate and carmustine are among the most common related causative agents.

COP (cryptogenic organizing pneumonia) is commonly defined by patchy migratory consolidations, classically with a “reverse halo appearance” (atoll sign), predominantly with peribronchial and subpleural distribution in the lower lung zones and typically associated with nodules and perilobular fibrosis, describing “arches” (arcade-like sign). COP typically responds well to treatment withdrawal, and corticosteroids are occasionally still required. Bleomycin, methotrexate and cyclophosphamide can provoke this condition.

HP (hypersensitivity pneumonia) is constituted by small, numerous, round and poorly defined centrilobular nodules, widespread areas of ground-glass opacities and hypoattenuating areas persistent even in expiration due to air-trapping phenomena (classically giving an appearance known as the “head cheese sign”). Chemotherapy withdrawal may be resolutive. Bleomycin and methotrexate are potential etiologies.

**Figure 1:**
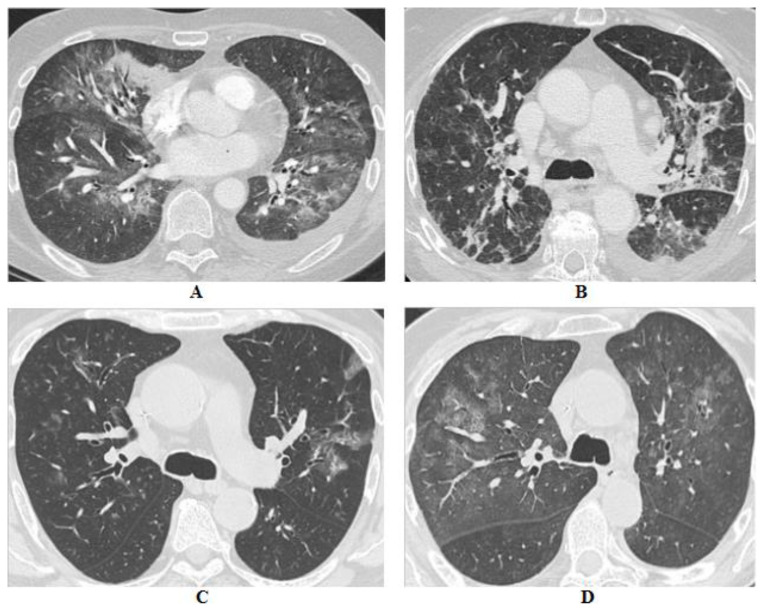
(**A**) 56-year-old male affected by renal cell carcinoma in treatment with everolimus, (**B**) 83-year-old female affected by esophageal adenocarcinoma in treatment with FOLFOX regimen, (**C**) 69-year-old male affected by gastric adenocarcinoma in treatment with FOLFIRI regimen, (**D**) 65-year-old female affected by pancreatic adenocarcinoma in treatment with gemcitabine. In (**A**,**B**) are shown multifocal consolidations, partly ground glass, in a pattern compatible with COP. In (**C**,**D**) scattered and patchy ground-glass opacities with crazy paving appearances, expression of alveolar damage, can be observed. All these patients were hospitalized during the COVID-19 pandemic, nevertheless, despite similar findings for both pairs, only for (**B**,**C**) was SARS-CoV-2 recognized as the causative agent, while for (**A**,**D**) chemotherapy was considered the most suspected etiology.

In comparison with the contingency COVID-19 pandemic, pneumonia typically presents with bilateral and peripheral ground-glass opacities accompanied by consolidations with typical interlobular septal thickening (“crazy paving” appearance). However, a broad spectrum of possible mimic patterns has been described, particularly including the aforementioned ILDs, especially COP and AIP [139]. See Figure 1.

**Figure 2:**
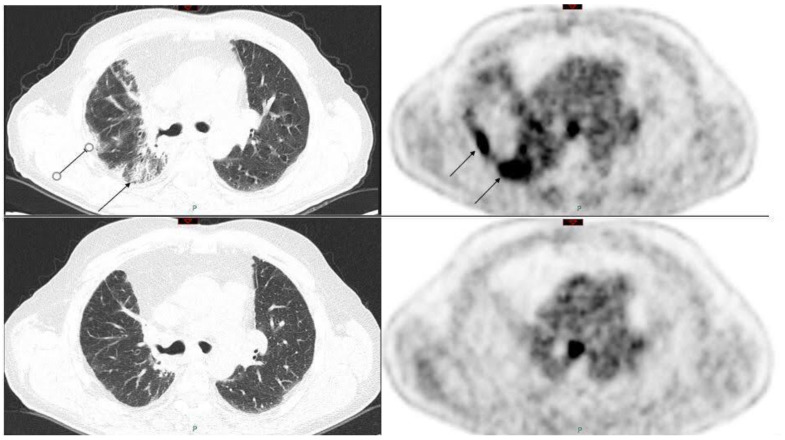
(**Upper row**): pre-therapy 18F-FDG PET/CT of a patient affected by lung cancer in treatment with atezolimumab, showing some areas of increased uptake of the radiopharmaceutical corresponding to mixed ground-glass and crazy paving lung CT alterations (black arrows). (**Lower row**): in PET/CT three months later, after atezolimumab suspension, no more areas of uptake are present, therefore indicative of an immune-related pneumonia.

Since the mechanism of uptake of 18F-Fluorodeoxyglucose (18F-FDG) is shared by neoplastic cells and inflammatory cells, false positive results interpreted as of oncological significance are frequently caused by underlying infections instead. Therefore, during several antineoplastic treatments, drug-related pneumonitis of any form may be erroneously interpreted as progressive disease by 18F-FDG PET/CT [140,141,142,143,144]. To date, this represents a hard to solve diagnostic dilemma, at least on the basis of the conventional image qualitative analysis. A possible help in this scenario could come from quantitative imaging, made possible from advent of machine learning [145,146,147]. We provide some examples of ILD images by 18F-FDG PET/CT in Figure 2.

About the clinical presentation, interstitial pneumonitis is often insidious. Dyspnea, dry cough, mild fever and fatigue may be the first clinical symptoms and signs. Differential diagnosis from other causes of pneumonia is essential.

The cornerstone of drug-induced pneumonitis treatment are corticosteroids, since the underlying mechanism of lung damage is the inflammatory and/or the immunological process. In severe cases, with the failure of high-dose steroid therapy, often the evolution to pulmonary fibrosis occurs, which may require other biological or immunosuppressive agents, such as cyclophosphamide, mycophenolate mofetil, azathioprine, infliximab, tocilizumab or rituximab [148,149]. In symptomatic disease, it is important to start treatment as early as possible, in order to achieve a better result.

The management of immunotherapy-related pneumonia, which is the most recently defined ILD, follows a specific guideline [150,151]. For non-immunotherapy related ILDs, if steroid therapy fails, the treatment is based on the therapy of connective tissue disease-related ILDs or idiopathic pulmonary fibrosis [149].

**Figure 3:**
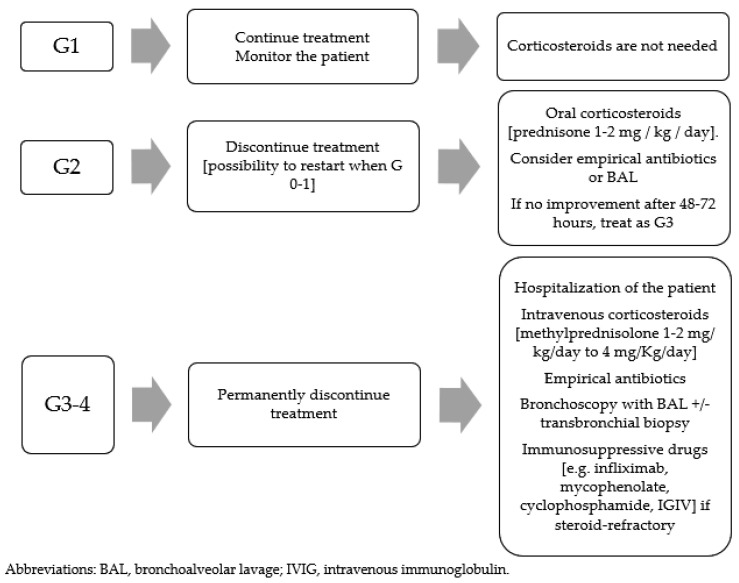
Management of pneumonitis according to severity.

The management of drug-related toxicity is based on its severity. Table 4 reports the classification of pneumonia according to severity and Figure 3 summarizes management according to grade, based on the American Society of Clinical Oncology (ASCO) guidelines for the management of immune-related pneumonia [150] (Table 4).

It could be identified as an association between the radiographic pattern and the clinical severity of pneumonitis, classifying the different patterns by toxicity grades, where AIP/ARDS pattern had the highest grade, followed by COP pattern, whereas NSIP pattern and HP pattern had lower grade [152]. It appears that NSIP and COP patterns respond better to treatment than UIP patterns [149].

Generally, steroid therapy is recommended for Grade 2 and higher, with oral prednisone 1–2 mg/kg/day. Grade 3 or 4 requires patient’s hospitalization and intravenous (IV) methylprednisolone 1 to 4 mg/kg/day. Steroid therapy should be continued until the event is resolved or improved to G1 and progressively reduced (e.g., 5–10 mg/week) and then stopped over at least 4–6 weeks, in order to avoid a rebound effect. This is very important in immunotherapy pulmonary toxicities, where, differently from other anticancer agents, it appears that flare pneumonitis can be more severe and extensive than the initial episode. Therefore, the gradual and slow tapering of corticosteroids is essential [152]. In severe cases, if there is no improvement after 48 h of high-dose steroids, it may be appropriate to add infliximab (an anti-TNF-a immunosuppressive agent) 5 mg/kg or mycophenolate mofetil 1 g twice a day or intravenous immunoglobulins (IVIG) 2 g/kg/day for 5 days, or cyclophosphamide. In these cases, a pneumological or immunological consultation could be indicated [150].

Patient’s immunosuppression (potentially caused by corticosteroids, oncological therapy, cancer, respiratory distress, poor performance status, etc.) requires the simultaneous administration of an empirical antibiotic prophylaxis to avoid superinfections, especially in Grades 3 and 4 (Figure 3).

In the case of prolonged steroid therapy (beyond 12 weeks), prophylaxis for opportunistic infections (e.g., trimethoprim/sulfamethoxozole for Pneumocystis jirovecii) should also be considered, just as calcium and vitamin D supplementation to avoid osteoporosis and proton pump inhibitors for gastric toxicity. The role of antifungal prophylaxis with fluconazole is less clear [150].

In addition, all other pharmacological and non-pharmacological symptomatic therapies, necessary for the management of the patient’s symptoms, must be adopted (e.g., oxygen therapy, therapy for cough, dyspnea, depression, anorexia, pain or any other potential associated symptoms and, of no lesser importance, pulmonary rehabilitation) [153].

## 6. Conclusions

The clinical manifestations of antineoplastic-induced ILD are not specific, and the diagnosis is determined on the basis of the exclusion of other causative factors. As not all oncological treatments bear the same risk as ILD, it is important to know the percentage of risk associated with the use of each drug. The mechanism underlying the toxicity in some cases could be linked to the mechanism of action of the drug itself and is mediated by the activation of the inflammatory-cytokine cascade. Therefore, the therapy consists of steroid anti-inflammatory treatment. Several radiological patterns are used to describe ILDs, unfortunately mostly without specificity to differentiate the multitude of potential conditions; however, radiology can be useful in grading the severity and guiding therapeutic management. There is no protocol to forecast this toxicity, but some patient baseline conditions may increase the risk of ILD. Differential diagnosis recently became even more complicated and crucial in the context of the arising awareness about the clinical respiratory manifestations of COVID-19. It is important to promptly recognize and treat this adverse event, which in some cases can be fatal.

## Figures and Tables

**Table 1:** Chemotherapeutic agents related to lung injury in the treatment of solid tumors.

Drug	Class	Incidence of Lung Toxicity (%) *	Main Patterns of Lung Toxicity
Bleomycin [16,17]	Antitumour antibiotics	10% ^1^	Interstitial pneumonitis Pulmonary fibrosis COP ^2^
Mitomycin C [18,19]		5–10%	Interstitial pneumonitis Pulmonary fibrosis PVOD ^3^
Anthracyclines [20]		Rare	Pulmonary fibrosis
Cyclophosphamide Ifosfamide [8,21]	Alkylating agents *Nitrogen mustards*	<1%	Interstitial pneumonitis Interstitial fibrosis Intraalveolar fibrosis Bilateral pleural thickening Pulmonary hypertension COP ^2^ Pneumothorax
Carmustine [22] Lomustine Fotemustine	*Nitrosoureas*	10–30%	
Temozolomide [23,24]	*Triazene compounds*	<1% ^4^	
Cisplatin [25]	Platinoids	Rare	Eosinophilic pneumonia
Carboplatin [26]		Rare	Pulmonary fibrosis
Oxalplatin [27,28]		<1% (0–4%)	Interstitial pneumonitis Pulmonary fibrosis COP ^2^
Methotrexate [9,10,11]	Antimetabolites	3–4% (up to 10% in arthritis patients)	Hypersensitivity pneumonitis Obliterating bronchiolitis Intraalveolar fibrosis
Pemetrexed [29,30]		Rare	Interstitial pneumonitis
Gemcitabine [12,31,32]		1–4% (up to 20% if with taxanes)	Interstitial pneumonitis Interstitial lung fibrosis PVOD ^3^ Non-cardiogenic pulmonary oedema AIP/ARDS ^5^ Diffuse alveolar haemorrhage Pleural effusion ↓DLCO (normal FEV_1_ eFVC)
Topotecan [33]	Topoisomerase inhibitors	Rare	Diffuse alveolar damage Interstitial pneumonitis Obliterating bronchiolitis
Irinotecan [34,35,36]		1–2% (up to 20% in lung cancer with paclitaxel)	Interstitial pneumonitis
Etoposide [37]		Rare	Interstitial and alveolar infiltrates Pulmonary fibrosis
Paclitaxel [38,39] Docetaxel [13,40]	Antimicrotubule agents	1–5% in three-weekly schedule (up to 20% if with gemcitabine and up to 30% with radiotherapy)	Interstitial pneumonitis Hypersensitivity pneumonitis Pulmonary fibrosis AIP/ARDS ^5^ Pleural effusions (capillary leak syndrome, especially for docetaxel)
Vinorelbine [41]		Rare	Interstitial pneumonitis

* Information regarding pivotal clinical trials or summary of drug/product characteristics is obtained from the literature. Information can be very different, depending on the sources, i.e., the criteria used to define pulmonary toxicity (clinical, radiological, etc.), the type of scheme in which the drug is contained, or the presence of concomitant radiotherapy and the type of patients and diseases analyzed (presence of possible risk factors for major lung damage). It was occasionally not possible to estimate a realistic incidence given the rarity of the event (mostly case reports) and because the drug is typically used in combination with other potentially toxic treatments. Therefore, the true incidence is often unknown, and we can only say that it is a rare/uncommon effect. ^1^ The incidence can be very different depending on the treatment regimen, i.e., from 5% to 16% in germ cell tumors treated with BEP (bleomycin, etoposide, cisplatin) or CVB (cisplatin, vinblastine, bleomycin) and from 10% to 53% in Hodgkin’s lymphomas treated with ABVD (doxorubicin, bleomycin, vinblastine, dacarbazine). ^2^ COP = Cryptogenic Organizing Pneumonia ^3^ PVOD = Pulmonary veno-occlusive disease ^4^ In gliomas, the incidence of nitrosourea-mediated lung toxicity is probably lower due to the low cumulative doses that are reached or the early progression of disease/death. ^5^ AIP = Acute Interstitial Pneumonia; ARDS = Acute Respiratory Distress Syndrome.

**Table 2:** Incidence of pneumonitis with targeted cancer therapies approved by the FDA and EMA in the treatment of solid tumors.

Class		Drug	Use		Incidence of Lung Toxicity (%), Any Grade *
TKIs (Tyrosine Kinase Inhibitors)	EGFR inhibitors	Gefitinib [44,45]	EGFR-mutated advanced NSCLC		1.6%
		Erlotinib [44,45]	EGFR-mutated advanced NSCLC		0.8–1.6%
			Advanced pancreatic cancer (+Gemcitabine)		1.6–2.5%
		Afatinib [44,45]	EGFR-mutated advanced NSCLC		0.7–1.6%
		Osimertinib [46]	EGFR-mutated advanced NSCLC EGFR-T790M-mutated advanced NSCLC		4%
	ALK inhibitors	Crizotinib [47,48,49]	ALK- and ROS-1-positive advanced NSCLC		1.2–1.8%
		Ceritinib [47]	ALK-positive advanced NSCLC		1.1%
		Alectinib [47]	ALK-positive advanced NSCLC		2.6%
		Lorlatinib [47]	ALK positive advanced NSCLC		1.8%
		Brigatinib [50,51]	ALK-positive advanced NSCLC		4.5–7%
	HER2 inhibitors	Lapatinib [52]	HER2-positive advanced BC (+Capecitabine/Trastuzumb/AI)		0.2%
		Tucatinib [53]	HER2-positive advanced BC (+Capecitabine + Trastuzumab)		1.2%
		Neratinib [54]	HER2- and HR-positive BC, adjuvant setting		0.07–0.1%
	Multikinase and angiogenesis inhibitors	Sorafenib [55]	Advanced HCC Advanced RCC Advanced differentiated thyroid carcinoma		Rare
		Sunitinib [56]	Advanced GIST Advanced RCC Advanced pancreatic NET		Rare
		Pazopanib [57]	Advanced RCC Selective subtypes of advanced STS		Rare
		Imatinib [58]	Kit-positive GIST, advanced and adjuvant setting Advanced dermatofibrosarcoma protuberans		Rare
	BRAF and MEK Inh.	Trametinib [59]	V600 BRAF-mutated advanced melanoma		2.4%
		Trametinib + Dabrafenib [60,61]	V600 BRAF-mutated advanced melanoma		≤1%
	PI3K Inh.	Alpelisib [62]	HR-positive HER2-negative advanced BC with a PIK3CA mutation, plus fulvestrant, second line		0.7–1.8%
mTORs inhibitors		Everolimus [63,64,65,66]	HR-positive advanced BC		12–38%
			Advanced RCC		14%
			Advanced NET		12%
			Advanced pancreatic NET		17%
		Temsirolimus [63,67,68]	Advanced RCC		2–22%
Monoclonal antibodies		HER2 inhibitors	Trastuzumab [69,70,71]	HER2-positive BC	0.6%
			Pertuzumab [72]	HER2-positive BC	<1%
		EGFR inhibitors	Cetuximab [73,74]	RAS wt advanced CRC Advanced squamous HN cancer	1%
			Panitumumab [75]	RAS wt advanced CRC	Case report
		VEGF inhibitors	Bevacizumab [76]	Advanced CRC Advanced BC Advanced NSCLC Advanced RCC Advanced ovarian cancer Advanced uterine cervix cancer	Case report
		VEGFR2 inhibitors	Ramucirumab [77,78,79]	Advanced gastric and gastro-oesophageal cancer (alone or with paclitaxel)	1.5% plus paclitaxe l0.4% alone
ADC		Trastuzumab emtansine [80]		HER2-positive BC	9%
		Enfortumab vedotin [81]		Advanced urothelial cancer	<1%
		Sacituzumab govitecan [82]		Triple-negative advanced BC	Unknown
		Trastuzumab deruxtecan [83,84]		HER2-positive BC	13.6–17.4%

* The incidence of pulmonary toxicity is derived from pivotal studies and the EMA summary of product characteristics. EGFR: epidermal growth factor receptor, NSCLC: non-small cell lung cancer, G: grade, ILD: interstitial lung disease, AEs: adverse events, ARDS: acute respiratory distress syndrome, ALK: anaplastic lymphoma kinase, ROS-1: reactive oxygen species, HER2: human epidermal growth factor receptor 2, BC: breast cancer, AI: aromatase inhibitors, HR: hormone receptors, HCC: hepatocellular carcinoma, RCC: renal cell carcinoma, STS: soft-tissue sarcoma, GIST: gastro-intestinal stromal tumor, NET: neuroendocrine tumor, CRC: colorectal cancer, HN: head and neck cancer, wt: wild type, VEGF: vascular endothelial growth factor, ADC: Antibody-drug conjugate.

**Table 3:** Incidence of pneumonitis with immune checkpoint inhibitors approved by the FDA and EMA in the treatment of solid tumors.

Drug	Indications	% of Pneumonitis (Including ILD), Any Grade
Nivolumab monotherapy [103,104,105,106,107,108,109]	Metastatic melanoma	1.5%
	Adjuvant melanoma	1.3%
	Squamous NSCLC second line	5%
	Non squamous NSCLC second line	3%
	HNSCC second line	2.1%
	RCC second line	4%
	Metastatic urothelial carcinoma after platinum cht	3%
Nivolumab + ipilimumab [110,111]	Metastatic melanoma	6.4%
	RCC first line for intermediate-high risk	6.2%
Pembrolizumab monotherapy [112,113,114,115,116,117,118,119]	HNSCC first line, PD-L1 positive with a CPS ≥ 1	6%
	HNSCC second line	4%
	NSCLC first line, PDL1 ≥ 50%	2.6%
	NSCLC second line, PDL1 ≥ 1%	5%
	Metastatic melanoma	1.8%
	Adjuvant stage III melanoma	3.3%
	Locally advanced/metastatic urothelial carcinoma second line	4.1%
	Locally advanced/metastatic urothelial carcinoma in adults not eligible for cisplatin-containing cht, PD-L1 positive and CPS ≥ 10	2%
Pembrolizumab + chemotherapy [112,120,121]	NCSLC first line in combination with carboplatin + pemetrexed	4.4%
	NCSLC first line in combination with carboplatin + paclitaxel/nab-paclitaxel	6.5%
	HNSCC first line in combination with platinum and 5-fluorouracil, PD-L1 positive and CPS ≥1	5%
Pembrolizumab + axitinib [122]	RCC first line	2.8%
Atezolizumab monotherapy [123,124]	Locally advanced/metastatic urothelial carcinoma second line	2%
	NSCLC second line	1%
Atezolizumab + nab-paclitaxel [125]	Advanced TNBC first line	3.1%
Durvalumab monotherapy [126]	Locally advanced unresectable NSCLC, PD-L1 ≥ 1%, not progressed following platinum based cht	12.6%
Durvalumab + platinum-etoposide [127]	Extensive-stage SCLC first line	3%
Avelumab monotherapy [128]	Metastatic Merkel cell carcinoma	1%
Avelumab + axitinib [129]	Advanced RCC first line	0.6%
Ipilimumab monotherapy [130,131]	Advanced melanoma	2%

ILD: interstitial lung disease; NSCLC: non-small cell lung cancer; HNSCC: head and neck squamous cell carcinoma; RCC: renal cell carcinoma; CHT: chemotherapy; CPS: combined positive score; TNBC: triple-negative breast cancer; SCLC: small cell lung cancer.

**Table 4:** Pneumonitis severity classification according to NCI-CTCAE version 5.0 and American Society of Clinical Oncology (ASCO) 2018 guidelines.

Guidelines	G1	G2	G3	G4
CTCAE Version 5.0	Asymptomatic, clinical or diagnostic observations only, intervention not indicated	Symptomatic, medical intervention indicated, limiting instrumental ADL	Severe symptoms, limiting self-care ADL, oxygen indicated	Life-threatening respiratory Compromise, urgent intervention indicated (e.g., tracheotomy or intubation)
ASCO guidelines	Asymptomatic, confined to one lobe of the lung or <25% of lung parenchyma, clinical or diagnostic observations only	Symptomatic, involves more than one lobe of the lung or 25–50% of lung parenchyma, medical intervention indicated, limiting instrumental ADL	Severe symptoms, hospitalization required, involves all lung lobes or >50% of lung parenchyma, limiting self-care ADL, oxygen indicated	Life-threatening respiratory compromise, urgent Intervention indicated (intubation)

Abbreviation: NCI: National Cancer Institute; CTCAE: Common Terminology Criteria for Adverse Events; G: grade; ADL: activity of daily life; Instrumental ADL: activities of daily life such as shopping, preparing food, using the telephone, managing money, etc.

## Data Availability

No new data were created or analyzed in this study. Data sharing is not applicable to this article.
